# Exploring the Unmet Needs of Teachers of Young Children with ADHD Symptoms: A Qualitative Study †

**DOI:** 10.3390/children11091053

**Published:** 2024-08-28

**Authors:** Reem Aldabbagh, David Daley, Kapil Sayal, Cris Glazebrook

**Affiliations:** 1 Special Education Department, Jeddah University, Jeddah 23218, Saudi Arabia; 2 NTU Psychology, School of Social Science, Nottingham Trent University, Nottingham NG1 4BU, UK; 3 Unit of Mental Health and Clinical Neuroscience, Institute of Mental Health, School of Medicine, University of Nottingham, Nottingham NG7 2RD, UK

**Keywords:** teachers, children, ADHD, training, themes, support, primary school

## Abstract

Background/Objectives: Children with Attention and Hyperactivity Deficit Disorder (ADHD) and those at risk of ADHD typically exhibit challenging behaviours that may disrupt the classroom environment and be frustrating for teachers. This study aimed to explore teachers’ experiences and emotions regarding teaching children with high levels of ADHD symptoms and their perceptions of what might help to meet their unmet support needs. Methods: Semi-structured interviews were conducted with 17 primary educational practitioners for children aged between four and eight years in the UK. Interview scripts were analysed using reflexive thematic analysis. Six main themes and 8 subthemes were developed. These included: (1) ADHD behaviours can disrupt the learning environment; (2) teachers face practical demands on their expertise and particular skills; (3) managing ADHD behaviours can be overwhelming for teachers; (4) teachers and children may treat children with ADHD negatively, which can impact on children’s emotions and lead to labelling; (5) existing support for teachers is limited; and (6) teachers need more specific training about ADHD. Results: The analysis revealed that teachers working with children with externalising behaviours such as ADHD can feel overwhelmed. Conclusions: The findings from this study suggest that teachers require more training in managing externalising behaviour in the classroom.

## 1. Introduction

Children with ADHD symptoms typically demonstrate challenging behaviours in the classroom setting, such as hyperactivity, fidgeting or squirming, and inability to remain focused. Children with ADHD symptoms can also show impulsivity, inattention, noncompliance, and aggression and are more likely to have other disruptive externalising behaviours, such as jumping, climbing, excessive talking, fighting and shouting. Carelessness and lack of concentration are also typical difficulties experienced by children with ADHD symptoms [1]. With a prevalence of around 5% [2] one or two children in every classroom are likely to have ADHD, with another 5% of children exhibiting some symptoms, but not meeting the threshold for diagnosis [3]. ADHD symptoms can directly impact children’s academic achievement [4], and teachers’ wellbeing [5] and become a focus for classroom disruption [6].

Typically developing children can also exhibit some behaviours that might look like ADHD. However, they gradually gain control over their behaviours, which in turn signals their readiness for school entry [7]. In contrast, children who are at risk of ADHD tend to maintain said behaviours as they enter primary school and often meet diagnostic criteria for the disorder [8]. Additionally, in many countries, summer-born children are the youngest in the classroom, with poorer control and attention caused by their age rather than ADHD risk [9]. Some children with ADHD symptoms have other health disadvantages that might increase their risk of developmental delay such as prematurity [10]. ADHD is also highly comorbid with externalising [11], and internalising disorders [12] that can impact educational achievement [13,14]. Thus, teachers in their classrooms may neglect children with high levels of ADHD symptoms who need additional support to enable them to reach their potential. Improved knowledge about better supporting children with ADHD in the classroom might benefit both children and teachers.

### 1.1. Impact of Symptoms on Teachers’ Wellbeing

Children spend more than 14,000 h of their lives in school, particularly in the classroom with their teachers [15,16]. Teachers play an essential role in forming a conducive and positive classroom environment [15,17]. However, teachers often suffer from stress and negative emotions due to various work-related factors [17,18,19].

Herman et al. (2020), introduced the 3C Theory of Teacher Stress, which explains the three pathways that contribute to building teachers’ stress: coping skills, competence in their practice and school system, and the administrative context in supporting teachers. This model links students’ disruptive behaviour to teachers’ stress [20]. Conversely, students’ disruptive behaviours can be one of the main factors that may cause stress (Liu & Onwuegbuzie [21]). Thus, it can also negatively impact on teachers’ self-efficacy in relation to teaching and classroom management [22]. A tense or hostile classroom environment poses risks to children’s emotional and academic development [23], which can lead to a cycle of disadvantage for the teacher and the child.

Although teachers can make a vital contribution to children’s educational achievement [24], teachers usually struggle with supporting and educating children with ADHD symptoms [25] due to the classroom disruption, which can often interrupt and interfere with the teaching process [6], and this can also affect teachers’ wellbeing [26]. Irrespective of gender, age, or teaching experience, teachers find children with ADHD symptoms challenging to teach compared with typically developing children [25]. A qualitative study in the US with teachers who had left teaching found that the challenges of maintaining discipline and handling challenging behaviour were perceived as significant factors contributing to their decision not to return to teaching [27]. A result of a meta-analysis exploring the relationship between students’ misbehaviour and teachers’ burnout indicated a significant effect on teachers’ sense of personal accomplishment, depersonalisation, and emotional exhaustion [26].

### 1.2. Teacher-Student and Peer Relationship

According to attachment theory [28], children may avoid developing externalising problems if they have stable relationships with their primary carers [29] which reflects on their academic achievement [30,31]. Teachers can provide a sense of security for students and play as an ad hoc or quasi-attachment figure which is vital for their development and well-being in the school setting [32]. Thus, it is crucial to secure positive teacher-child relationships to provide children with ADHD symptoms with a positive school environment [33]. Children with behavioural problems can face social exclusion in school and may struggle to form meaningful relationships with peers [34,35], and they may thus suffer from loneliness [36]. Teachers’ role is essential since they have the ability to shape social interactions among students in the classroom dynamics, serving as an “invisible hand” [37]. Endedijk et al., (2021) conducted a meta-analysis of 287 studies to investigate the link between teacher-child relationships and peer relationships. The findings show that conflicts between teachers and students in school settings can predict negative peer relationships, and vice versa. The findings suggest that reducing problematic interactions with students can help students with behavioural problems build better social dynamics in school setting [38].

### 1.3. The Role of Stress on Functioning and Coping

The Transactional Model of Stress and Coping [39], is used in the present study to help explore teachers’ experiences and emotions regarding teaching children with high levels of ADHD symptoms and their perceptions of any unmet needs, as teachers face stress every day at work. According to this model, stress is an emotional response to stressors (demands) in the environment. When emotional, physical and cognitive demands surpass the limit of the practical and emotional resources available, stress develops and jeopardises the individual’s wellbeing. Primary appraisal of the situation is how the person cognitively perceives the external factors as a threat, or challenge. The secondary appraisal is judging the capability of the individual’s internal potential to cope or the external resources that can help in the situation. People may experience a different level of stress based on their appraisal of the stressor. The process of coping requires creating a balance between the external demands and the internal and external resources [39].

According to this model, the individual can attempt to manage stressors using two approaches. The first one is problem-focused coping, which aims to reduce demands, for example, developing skills to reduce the impact of disruptive behaviour in the classroom. Emotion-focused coping aims to minimise the perceived impact of the stressor using psychological techniques such as denial or by behavioural strategies such as alcohol use or exercise [39]. Balancing the demands placed on teachers with the practical and emotional resources to manage those demands can help in building resilience to stress to alleviate daily stress for teachers, while also ensuring a good learning environment for children.

### 1.4. Evidence for the Best Time to Deliver School-Based Interventions

Interventions aimed at preventing or alleviating behavioural problems before they develop or cause aggravation in the classroom are crucial in early years schools. Early school-based intervention to prevent the accumulating effects of stigma and educational deficits is key to breaking the cycle of disadvantage for children. According to the DSM-5TR, the minimum diagnostic age for ADHD is four years (American Psychiatric Association, (2013) [1]), which is the time when the risk usually emerges during the preschool period [40]. At the age of 7.8 years, children in the UK start Key Stage 2, a stage at which the school increasingly demands self-regulation skills from children [41]. Thus, the period from age four to eight years is a very crucial developmental phase that starts with the possibility of ADHD symptoms emerging [42] and the expectation of showing school readiness [7], and ends with the transition to Key Stage 2 [41]. Effective management of early emerging symptoms in young children with ADHD symptoms is important to prevent adverse social and academic effects [43].

This study aims to understand teachers’ experiences of teaching children with ADHD symptoms and their unmet needs for support using semi-structured interviews. It explores how supporting children with ADHD symptoms may affect teachers’ wellbeing, and what assistance they believe they need, alongside their current level of support. A greater understanding of these issues is essential to provide teachers with appropriate support to enhance their classroom management abilities for children with ADHD symptoms.

## 2. Materials and Methods

### 2.1. Design & Justification

This qualitative study uses semi-structured interviews to explore teachers’ experiences and unmet needs with regard to managing children at risk of ADHD. The study design allows in-depth exploration of teachers’ experiences when working with children with ADHD; how this can affect their wellbeing, what support they feel they need, and the level of support currently provided. This understanding is important to the development of interventions to enhance the management skills of teachers of children with ADHD symptoms in the classroom.

### 2.2. Materials

An interview schedule was developed in collaboration with an expert on ADHD interventions (DD). It was piloted with an experienced teacher and revised in line with the teacher’s comments. For example, in the pilot interview, we referred to children at risk of ADHD. According to the teacher, this term was confusing as she teaches children who are diagnosed with ADHD and others who only display ADHD symptoms. Accordingly, the term was changed to ‘children with ADHD symptoms’.

The semi-structured questions focused on teachers’ experience with children with ADHD symptoms, including their way of responding to their behaviours. The questions also focused on current resources for teachers that can support managing these behaviours, and explored any unmet needs that teachers described.

### 2.3. Recruitment

Recruitment was conducted by circulating an email to gatekeepers at Nottingham City Council schools with study advertisement and details about the aim of the study and the inclusion criteria. The advertisement was also included in weekly bulletins to all Special Educational Needs Coordinators SENCos at Nottingham City Council schools. The researcher also contacted headteachers in Nottingham, and London to circulate the advertisement to teachers. A total of twenty-four schools were contacted. Recruitment also included snowball sampling methods with participants asking them to pass on the study advertisement to colleagues.

### 2.4. Participants

The study used a purposive sampling technique via gatekeepers and snowball sampling to interview teachers of children aged between four and eight years with ADHD symptoms. Teachers were eligible for inclusion in the study if they currently or previously had experience of teaching children aged four to eight years old with ADHD symptoms. Teachers were recruited until data saturation was achieved.

### 2.5. Procedure

The researcher conducted the interviews from the 14 December 2018 until the 10 March 2019 individually in a quiet place. The interview appointments were held with the participants in their free time, 11 face-to-face and six via telephone. Written and verbal consent were obtained from all the participants either face to face or by email for the telephone interviews. All interviews were recorded using a digital audio recorder. Recorded interview files were transferred to a password-protected secured database using the University of Nottingham cloud. The interviews were transcribed verbatim by the researcher. Finally, the whole file was proofread and finalised. Any names and any personal identifiers were removed from the transcript. Each participant was assigned a code to ensure confidentiality and anonymity. A £10 electronic gift voucher was given or emailed to all participating teachers following the completion of the interview.

### 2.6. Ethical Approval

The study was approved by the Division of Psychiatry and Applied Psychology Research Ethic Committee on 10/10/2018 reference number (DPAP-2018-0127-2).

### 2.7. Reflexive Thematic Analysis

Braun and Clark developed six clear steps to follow when conducting Reflexive thematic analysis [44,45], which can help in making this methodology more replicable. The researcher should apply the steps to the data set to generate a systematic development of themes; at the same time, the approach can still guarantee flexibility in its epistemological position, by allowing the author to analyse and judge a large or small dataset collected from interviews, focus groups, surveys, diaries, films, or observations, then this data can be interpreted by themes using a clear process.

Reflexive thematic analysis can be inductive whereby the themes are derived from similarities that identified from the data, or deductive in which themes are driven by previous research findings or specific theories or predictions [44,45]. The analysis can also be on a latent level or on a semantic or manifest level. Latent level focuses on the implied or implicit meanings and interpretation for the data set [44,45]. The semantic or manifest level captures what can be derived from what has been explicitly stated in the data.

**Figure 1:**
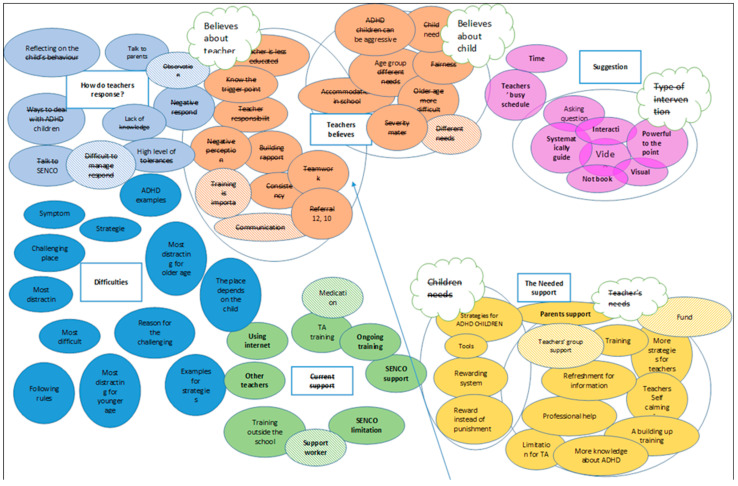
Code mapping.

In this study, reflexive thematic analysis was guided by the social constructivism paradigm. The study focus on finding and reporting consistent dataset patterns that are driven by the research question following the phased approach of Braun and Clark (2006, 2014) which are: familiarising oneself with the collected data, initial code identification (see Figure 1), theme development, theme reviewing, theme refining and naming, and generating the report. Reflexive thematic analysis was used inductively and aimed to capture meaning at the latent and manifest level seeking to identify recurrent concepts among transcripts [44,45].

### 2.8. Data Analysis

After interviewing 15 teachers, RA felt that no new patterns or information were being identified in the interviews that could generate new themes. Two more teachers were interviewed to ensure that no new information could be obtained. After that, data saturation was considered adequate and the interviews ceased [46,47].

The data were analysed using the six steps of reflexive athematic analysis using the Braun & Clarke (2019) method. First, the interview and transcribed verbatim. Then the transcripts were read several times to gain a broad understanding of the content of the interviews. The whole process was carried out by the same researcher (RA) who conducted the interview and spent time with the participants before and/or after the interviews. Then the researcher engaged with the data through transcription and reviewing the interview notes in order to guarantee prolonged immersion in the data. Secondly, the initial codes were identified. The data were grouped using meaningful codes that were specific and helped to develop the themes in the following step. The codes were checked in an organised way using Excel. Reflective journals were kept throughout the entire data collecting, coding, and analysis processes. Peer debriefing was also used, while member checking was conducted by sending three teachers their scripts to check the coding to ensure credibility. Third, a broader meaning for the codes was created and presented in themes. The initial themes were checked by team members (RA), (CG), (DD) & (KS). Fourth, a thematic map was developed through the researchers having a deep understanding of the themes. Mind mapping was used to connect the themes and make sense of their relationships and keep track of the evolution of the themes. This helped in reviewing the themes and reaching a thorough interpretation. Peer debriefing and member checking was also used in this step. Fifth, the themes were refined and identified by name. Subthemes were also identified. Then the themes were checked by an external auditor to gather constructive feedback. Feedback was also gathered by presenting the themes at the Centre for ADHD and Neurodevelopmental Disorders Across the Lifespan conference (CANDAL). Finally, the report was written including the final themes and subthemes and textual extracts to illustrate how themes were selected. Negative case analysis was also applied to the analysis, which involved investigating opinions and arguing against the meaning interpreted from the data. Excel was used in the analysis to facilitate the ongoing use of reflective journals throughout the whole process of data collection, coding and analysing. Example quotes from the scripts were provided for each theme, as recommended by Consolidated Criteria for Reporting Qualitative research (COREQ) for reporting qualitative research [48], and the American Psychological Association [49] to provide the analysis with transparency [50].

### 2.9. Trustworthiness

Qualitative research is inherently subjective, and findings can be influenced by researcher bias. The researcher must ensure trustworthiness in order to minimize bias [51]. The theoretical underpinning for this study is social constructivism. The social constructivism theory focuses on understanding how people create their social realities in their environment by interpreting the constructed reality [52]. Constructivism acknowledges that the researcher’s background and social interpretation can influence the constructed reality. It thus aims to minimise that influence and ensure trustworthiness by adopting a reflexive approach where researchers reflect on their experiences and values [53].

The Consolidated Criteria for Reporting Qualitative Research checklist (COREQ) were followed while conducting the qualitative research [48]. This checklist covers three main domains: Research Team and Reflexivity; Study Design; and Analysis and Findings including 32 criteria. Using this checklist throughout conducting qualitative research is suggested to enhance transparency in qualitative research [48]. Age, years of experience and ethnicity were not recorded to provide teachers with sense of anonymity, however, it could be considered a limitation in this study.

## 3. Results

**Figure 2:**
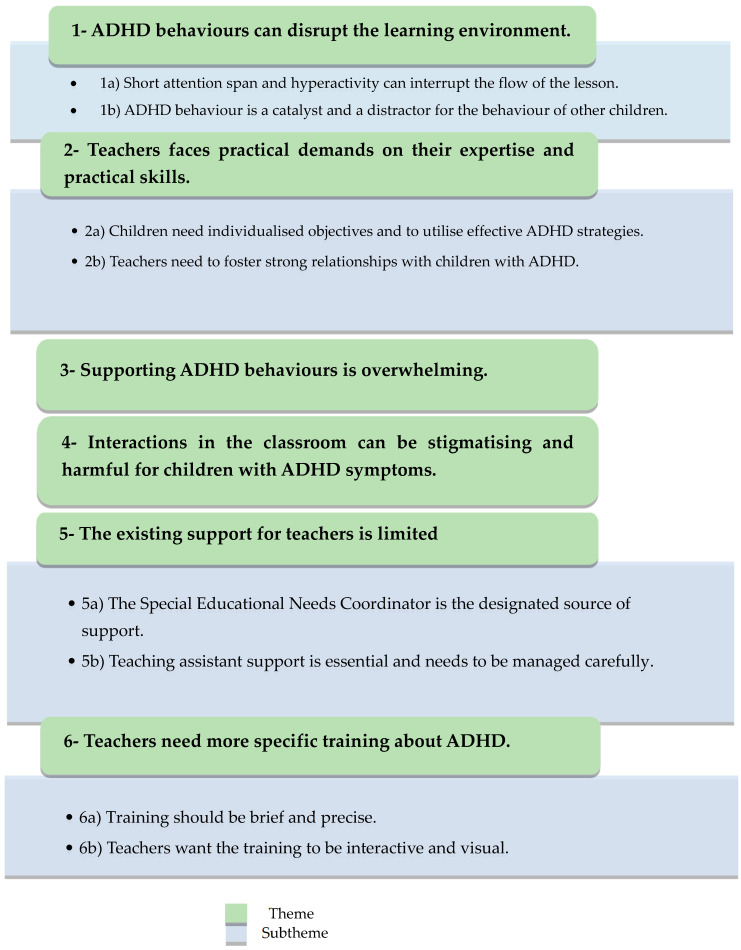
Summary of the overarching themes and subthemes [54].

In total, 17 teachers from nine schools were recruited for the study. Nine teachers were from faith schools, eight of which were Muslim schools, and one was from a Church of England school. The rest of the teachers were from secular schools. The participants’ role at the school varied; three were teaching assistants and two were Special Educational Needs Coordinators (SENCos), and the rest were teachers. One of the participants was male. Six primary themes and eight subthemes were identified from the data. The themes are summarized in Figure 2.

### 3.1. ADHD Behaviours Can Disrupt the Learning Environment

It was evident from the analysis that ADHD behaviour management is challenging for teachers. Teachers commented on children’s ADHD-type behaviours in the classroom and the disruption caused due to such behaviours.

#### 3.1.1. Short Attention Span and Hyperactivity Can Interrupt the Flow of the Lesson

Teachers highlighted that children with ADHD exhibit a range of behaviour that can have a negative impact on the classroom. Teachers identified poor attention as the most challenging aspect of children’s behaviour. The child with ADHD symptoms can be easily distracted and go off-task, which results in an inability to finish their work sufficiently. A poor attention span can also negatively impact on their learning process. Almost all the teachers in this study described the difficulties they face with inattentiveness in children with ADHD symptoms.

T7: “Another challenging thing is getting them to focus on a task for a required period of time. I’m teaching the early years, The tasks we do are already really short, but some children are not even able to focus for a really short time… And so, they kind of jump from one thing to another”.

T10: “Lack of concentration, moving in the classroom, lack of focus whenever we are sat on the carpet. Like if we are explaining something, they are in a different world”.

Not only can these symptoms distract their learning, but they can also distract other children’s learning and concentration. Children with ADHD symptoms can easily go off-task and find another thing to do, like fidgeting and squirming, chatting, or even leaving their place, which can act as a distraction for other children. Teachers stated that other children kept complaining about not being able to focus because of the behaviours of the child with ADHD symptoms.

T10: “If they can’t sit, they move about in the classroom. They’ll be distracting other children”.

T12: “If they’ve gone off task, or their focus is not sustained, so therefore that is when they start fidgeting or making a noise, which is distracting to others. They quite often say: he’s “moving tables”, “I can’t work!” Or, “He’s tapping the pencil or making a noise” and stuff like that. So, as well, you have to be careful where you position them in the classroom”.

#### 3.1.2. ADHD Behaviour Is a Catalyst and a Distractor for the Behaviour of Other Children

Teachers commented that ADHD behaviour in young children may sometimes appear provoking to their peers. They may imitate these behaviours, which can add more load to the classroom and cause distraction.

T1: “I’ve also seen children with ADHD in the younger years to be quite popular, because it seems like this is quite interesting”.

T7: “And then they start to play the same way, and it’s like, oh my goodness, I don’t want this to happen with you as well. I mean one is enough to deal with”.

Two teachers also stated that, in contrast, other children might help the teacher in managing the behaviour of children with ADHD by reminding them of the classroom rules and assisting them in accomplishing academic tasks.

T2: “You’ll find that they will start helping them anyway, and they are all saying that [name] (whispering), ‘You need to be quiet!’, and saying to them (whispering), ‘No don’t do that! don’t do that!’ (Laughing)”.

### 3.2. Teachers Face Practical Demands on Their Expertise and Practical Skills

#### 3.2.1. Children Need Individualised Objectives and to Use Effective ADHD Strategies

Teachers highlighted that children with ADHD symptoms have different needs from typically developing children in the classroom. Some of the teachers described them as “demanding”. It was also noticed that many the teachers had agreed on the efficacy of one-to-one lessons or small group activities.

T9: “We are doing a plan, especially for him, even if you are teaching or if you are writing the success criteria for the whole students. His own success criteria are not the same, so we must go to his level”.

Teachers explained how they are constantly modifying the curriculum in a way that can fit the children’s ability and their level of attentiveness. They might deliver their instruction in a different way or steer their resources towards meeting the children’s needs. They may design a particular activity or tailor tasks specifically for those children.

T12: “I think it is more strategy of what and how you can tailor the curriculum or how you can reach learning strategies for the child and help them the most”.

T15: “I might completely differentiate the work I might need to arrange for my classroom assistant to start them off on a task or monitor them”.

#### 3.2.2. Teachers Need to Foster Strong Relationships with Children with ADHD

Teachers reported that communicating with the child is important if learning goals are to be achieved, because they often lack concentration and cannot follow instructions easily. Thus, clear strategies and rules for the classroom often help.

T2: “You need to give them mechanisms for being able to ask you if they feel they don’t know what they’re doing, because that can frustrate them if they don’t, as they can’t process a whole set of instructions, so, if they get halfway through and don’t know what to do, you need to make sure that they know a safe way of dealing with that rather than carrying on and not knowing what to do”.

Building a rapport and having a warm and encouraging environment was stated as a significant approach when supporting children with ADHD symptoms and this requires effort.

T1: “I like to try to greet every single child, and I try to engage their emotions, I talk to parents to see if there are any sort of worries as if a child is a bit teary in the morning, they can take it with them the rest of the day.”

One of the teachers highlighted the need to teach children emotional regulation and strategies to communicate their emotions.

T15: “We try and teach them how to manage those feelings and how they show their emotions and moving from feeling sad to feeling happy and smiling”.

Not only do teachers strive to build a friendly relationship with the child, but they also have to reflect on the child’s behaviours and the factors that can trigger such behaviours or maintain them. Teachers also stated,

T17: “Every child is different, and every situation is different as well. It’s all about trial and error and thinking about the child and their individual traits and what is going to suit their needs the best”.

Teachers thought that children with ADHD symptoms need to learn that there will be implications for their behaviour. Teachers emphasised the vital role of having a reward system as a handy tool to be used with children with ADHD symptoms. However, consistency in supporting the child is very important yet challenging. It was suggested that teachers can replace punishment with a reward system, which may result in a better outcome.

T5: “I think strategies, instead of labelling them or giving them detentions or threatening them with ‘I will give you this, I’m gonna give you a yellow card, I wanna give you detention, I’m gonna ring your mom, so take away negativity and put it into positive reinforcement, so the child changes their behaviour to positive’”.

### 3.3. Supporting ADHD Behaviours Is Overwhelming

Teachers described children with ADHD symptoms as demanding, requiring attention, and challenging. The situations that teachers face with these children are stressful and can result in frustration and helplessness. Some teachers thought it can affect the wellbeing of teachers and lead to reluctance to work. It was clear that teachers described their stress differently. Comments in the interviews about having children with ADHD behaviour in the classroom revealed the teachers’ different levels of predicament. For example, these comments were identified from different teachers

“It’s a nightmare!”; “I want to pull my hair!”; “I am gonna shut the door, and I’ll have a little scream, and then, I’m going to spin a smile in again!”

Others described the situation as emotionally exhausting.

“It makes me feel quite sad”; “It’s upsetting me to see that I can’t do anything to stop that child”; “It’s still a challenge every day”; “It’s stressful”; “It’s frustrating, and we’re all human beings and sometimes we get a bit tired”.

TA11: “So, it can cause a lot of stress because we are just non-stop trying to make sure that the class is a safe environment for everybody else”.

While acknowledging the pressures, one teacher valued the stimulus to learn new strategies.

T7: “It’s frustrating, but it also makes you want to learn more about how to deal with that child; so, it’s kind of a good thing as well, because it opens up, you have to go and do some reading, you have to go and do the research, reach out other professionals as well, so yeah, it’s quite stressful”.

Only one teacher believed that having children with ADHD symptoms in the classroom does not have any impact on teachers.

T2: “I don’t think that there is much of an impact on teachers. If you don’t let it have an impact, it wouldn’t cause you stress”.

Another teacher also pointed to self-regulation and self-calming as a vital technique for teachers to acquire to cope with the teaching stress.

T4: “If you have some strategies on how to control yourself… that would be good”.

### 3.4. Interactions in the Classroom Can Be Stigmatising and Harmful for Children with ADHD Symptoms

Teachers described a range of ways in which the child’s behaviour can have negative repercussions for the child. They. also said that when negative responses occur in the classroom, children can be labelled by their peers and teachers as “naughty” or as having ADHD with or without diagnosis.

T5: “It brings out negativity in teachers”.

T1: “Some teachers and some children just say that the child is ‘naughty’. It becomes a label for some children with ADHD”

Challenging behaviours can also cause teachers to avoid working with that child.

T8: “It’s easy to push it away from you as a teacher and make it someone else’s responsibility”.

TA11: “Most of the time the teacher would allocate the Teaching Assistant to work with those children”.

One teacher described the ways in which the behaviour disrupted peer relationships, with the child being blamed for any disruption in the classroom.

T14: “They use it, and they abuse it, they’ll blame the child and then use the child as a scapegoat”.

Teachers also acknowledged that the child with ADHD symptoms can be subject to restrictions, which can impact on their learning and adjustment at school. The child can also receive a lot of criticism or restrictive practices.

T4: “They can be really angry with a child, and then it’s like, ‘No play for you today…’ But it’s really not justifiable, that can affect him for the whole day and he, becomes completely depressed, he can’t do any work. He can’t do anything”.

One teacher pointed out the effect of labelling on the child, and how teachers perceive the ADHD label as being naughty.

T16: “There are some teachers, if ADHD is identified, they may think that the child is, I wouldn’t say naughty, but, not listening? Whereas there could be an actual underlying cause like ADHD”.

One teacher suggested that sometimes the behaviour might be triggered by the teacher, resulting in a reaction from the child.

T5: “Even if they have a very busy schedule, I think we should put in like a morning meeting or an after-school meeting or something in place, If once a week or one task a day, to understand why children’s behaviour may escalate, or if teachers provoke it as well”.

### 3.5. Existing Support for Teachers Is Limited

The Teaching Assistant, Special Educational Needs Coordinator and training can support teachers, but there are some limitations.

#### 3.5.1. The Special Educational Needs Coordinator Is the Designated Source of Support

Teachers referred to the Special Educational Needs Coordinator (SENCo) as the first-person teachers seek for help. They observe the children, identify their needs and then develop an Individualised Education Plan (IEP) for the child and help the teacher to manage their behaviour.

T1: “For example, our SENCo would develop a plan with teachers and with people who had worked with that child before to sort of develop a behaviour plan”.

Although SENCos are usually involved with the teacher to help children with ADHD symptoms, some teachers mentioned conflicting demands placed on SENCos and that they may not always have the time to help teachers in supporting children with challenging behaviours.

T13: “You know, you have your SENCo in school, and they are generally very good in little ways. it can be like, ‘Oh no! You’ll find a way.’ You know.”

TA11: “Usually, it is easier to chat with other teachers…You could get hold of them a lot easier than the SENCo because at that moment the SENCo would actually be deputy head, so she’s got two hats to wear”.

#### 3.5.2. Teaching Assistant Support Is Essential and Needs to Be Managed Carefully

Many teachers complained about the lack of teaching assistant support, such as moving them from one class to another. They thought that having the teaching assistant support in the classroom is crucial for those children, as the teacher needs to concentrate on delivering a good lesson. Teachers sometimes need time out to calm down before coming back to their classroom, but if they are the only adult in the classroom, it is impossible to benefit from such a break, which can affect their wellbeing.

T17: “Support is needed for teachers in these schools where funding is being cut all the time, and there’s not necessarily teaching assistants in every class anymore. It can be really hard for that teacher and their mental health and their wellbeing as well. Sometimes, they need a minute to just go and cool down and come back and address the situation. But when you are the only one in the room and there is nobody to come and help you, then yeah, there is nobody to come and help”.

One of the teachers said that passing the child’s responsibility completely to the teaching assistant may result in the child being a dependent learner, whereby the child gets used to having an adult around.

T1: “It’s really important to have a good understanding of ADHD because it’s very easy if you get support in your class, and push that support on them, and then they lose their independence and become a dependent child”.

One of the Teaching Assistants mentioned that she had to work with a child with ADHD most of the time, which limited her availability for other children who were also in need of support.

T11: “I’m a teaching assistant, so most of the time the teacher would allocate the Teaching Assistant to work with those children. So, to be honest, as a teaching assistant, I would have most of the responsibility to try and calm them down by sitting with them, speaking to them, or doing the exercises that we have to do in the class with them. So, it takes my role to assist the other children in the classroom into providing assistance one-to-one”.

### 3.6. Teachers Need More Specific Training about ADHD

Teachers pointed out the need for more training and professional help regarding classroom management. Similarly, the need for more knowledge about ADHD was accentuated in the analysis. Teachers need to identify the signs of ADHD, in addition to learning more techniques to manage the behaviour of children with ADHD symptoms and the whole classroom.

T11: “I’d say training, training, training is a must for everybody that works with children because you never know when you have those challenges or these problems in classrooms”.

Ongoing training is vital to keep building up knowledge and professional development. Regardless of teachers’ tiring schedules, they pointed out to the need for acquiring more knowledge about ADHD.

#### 3.6.1. Training Should Be Brief and Precise

Teachers acknowledged that they have a very busy and tiring schedule and therefore the intervention should be brief and precise.

T13: “At the end of the day the brain capacity is gone. So, the training should be brief…”

One teacher suggested having the intervention in her spare time.

T10: “Maybe at the weekend, if you leave it (the training) flexible to the teacher because, you know, teachers are really overloaded with work”.

#### 3.6.2. Teachers Want the Training to Be Interactive and Visual

Teachers suggested that visual aids can achieve the goal of the training since they can engage teachers who can easily get bored or overwhelmed at the end of the school day. They need strategies that can work and information that is powerful and to the point. Some teachers also suggested having interactive training. They believed that asking questions may clarify ideas. Teachers would also like to have the opportunity to talk to professionals and ask about some specific cases.

T13: “I haven’t got the time to sit and read a book after I’ve marked twenty-six maths books. There’s got to be something quick, videos are good because they are quickly accessible. Whereas if you put a book plan, you think really “I’ve had a day and a half”.

T7: “I think more interactive kinds of thing, I think for books you had enough of reading when you were at university”.

T4: “If there are counsellors that we can ask, ‘This is happening, what could I do?’ I think they can give some expert ideas on how to deal with these things”.

## 4. Discussion

Six main themes and eleven subthemes were developed. The six main themes were as follows: (1) ADHD behaviours can disrupt the learning environment; (2) Teachers are facing particular practical demands on their expertise and practical skills; (3) Supporting ADHD behaviours is overwhelming; (4) Interactions in the classroom can be stigmatising and harmful for children with ADHD symptoms; (5) Existing support for teachers is limited; and (6) Teachers need more specific training about ADHD.

In the analysis relating to Theme 1, “ADHD behaviours can disrupt the learning environment” teachers indicated that ADHD behaviours such as hyperactivity, inattention and impulsivity may interfere with the classroom, thereby interrupting the learning environment and lesson flow. These findings are in accordance with a meta-analysis that investigated the relationship between ADHD symptoms and associated functional impairment, with the findings indicating that hyperactivity and impulsivity may lead to classroom disruption [55]. Furthermore, ADHD behaviour is potentially a catalyst and distractor for other children’s behaviour in the classroom. This may be connected to and explained by other qualitative analysis by Ward et al. (2021), who investigated the optimal type of training required to support children with ADHD in primary school in the UK. According to that study, teachers think it is difficult for peers to accept the notion that a child in the classroom is being rewarded for basic behaviour [56]. Therefore, certain children might behave differently causing greater disruption in the classroom. Such classroom disruption might lead to other ramifications for both teachers and children with ADHD symptoms.

In the analysis pertaining to Theme 2, the teachers emphasised that they are facing demands on their skills and expertise. This is unsurprising, because according to The Teaching and Learning International Survey (TALIS) completed by 260,000 teachers from 15,000 different schools across 48 countries, over 60 per cent of teachers expressed dissatisfaction with the amount of time they spend managing classrooms with disruptive behaviours [57].

Further relating to Theme 2, teachers highlighted the need to try to foster strong relationships with children, recognising that this is particularly crucial and can assist with managing students’ unacceptable behaviours, which may prove fruitful for both the child and the teacher. As proposed in a meta-analysis, a negative teacher-child relationship may undermine children’s school attainment and cause emotional and social problems for the child with ADHD [58]. Moreover, a negative teacher-child relationship can detrimentally affect teachers’ wellbeing over the long-term [59,60]. This suggests that training teachers is crucial to support both teachers and children with ADHD symptoms, Aldabbagh et al. (2022) in a meta-analysis, established that teacher-delivered interventions have positive effects in terms of reducing children’s behavioural problems, as well as enhancing teachers’ positive behaviours and relationships with children with ADHD symptoms [61].

It was evident from the strength of feeling in Theme 3 ‘Supporting ADHD behaviours is overwhelming’, that working with children with ADHD symptoms places significant emotional demands on teachers and exacerbates their stress levels. Teachers described their stress in various ways and with different phrases that indicated different levels of stress, while one teacher did not consider it stressful. The variation in teachers’ stress levels emphasises the strong correlation between each teacher’s coping capability and their tolerance of managing these behaviours, as explained by the 3C theory of the competent pathway [20]. Furthermore, the quotes indicate that teachers respond differently to such behaviours (demands) in accordance with the Transactional Model of Stress and Coping [39], with some attempting to apply emotional coping by regulating their feelings by reacting emotionally to problems. Meanwhile, others adopted problem-focused coping by actively working to solve problems.

The analysis shows how teachers can feel overwhelmed by ADHD behaviours. Consequently, children may be negatively affected by the powerless feeling of the teachers, thereby potentially indirectly leading the teacher to implement ill-considered strategies and labelling of children as being naughty, as discussed in Theme 4, ‘‘Interactions in the classroom can be stigmatising and harmful for children with ADHD symptoms’’. These findings are in accordance with research investigating Korean pre-service teachers’ perceptions of labelling, which confirmed that labelling may arise prior to and following diagnosis [62]. Stressed teachers may be less tolerant and more aggressive when working with students with ADHD symptoms, producing a negative impact on the student [63]. Additionally, it has been suggested that people label and stigmatise as a form of self-defence when feeling anxious or intimidated by a certain situation [64]. This can also be a form of emotion-focused coping in order to reduce negative appraisals of their teaching abilities [39]. This emphasises the crucial nature of fostering resilience in teachers to assist them with managing rising pressure and avoiding negative consequences for both teachers and students.

Additionally, this theme can be compared with a previous qualitative analysis of interviews with 13 students diagnosed with ADHD, where the results showed that students had indicated that certain teachers’ reactions may traumatise students and provoke their anger [65]. Stressed teachers may feel less patient when working with children, leading to unpleasant experiences for children [63]. Previous research has also determined that school setting complexity and routines may prompt ADHD behaviour and detrimentally affect the teacher-child relationship [66]. The previous point highlights that the teacher-child relationship is dyadic in nature, potentially causing a cycle of disadvantage. Increasing teachers’ understanding of the nature of ADHD and its impact on children, alongside effective classroom management strategies, is essential for creating classroom harmony.

Concerning Theme 5, “The existing support for teachers is limited” teachers stated that their access to specific training and resources is limited, although teachers appreciate the SENCo’s support. However, they pointed out that SENCos are usually wrestling with a burdensome workload, making them inaccessible. Previous research has indicated that a lack of training and resources is associated with higher levels of teacher stress (Agai-Demjaha [67,68]. Moreover, lower self-efficacy levels in relation to classroom behaviour management can predict higher levels of stress [69]. Interventions aiming to enhance teachers’ classroom management skills can provide a sense of capability, as well as reduce teachers’ stress [69,70,71]. It is crucial to balance between the demand on teachers and the available resources, to provide teachers with a sense of control over their work environment, thereby reducing stress. This is in accordance with the Transactional Model of Stress, which proposes that the level of stress experienced is affected by both perceived demands (cognitive appraisal) and resources, for example coping skills [72].

Given that certain teachers feel challenged by managing ADHD behaviours, it is noteworthy that all teachers emphasised their requirement of professional development specific to managing ADHD behaviour (Theme 6). Such findings are consistent with other studies which evidence that most teachers and teaching assistants would like greater training on managing children with ADHD [56,73,74]. Specifically, here, teachers requested that the training they receive should be precise and to the point due to them being overworked. Additionally, they emphasised the need for training to be available via videos or gadgets that they can use when convenient. Similarly, Ward et al. (2021) indicated that teachers need training to be accessible when required, as opposed to only during school hours [56]. Tracing back to the problem-focused approach from the Transactional Model of Stress [72], this enables teachers with the essential skills to benefit from a less disruptive classroom environment, which can strengthen their feelings of competency; this is considered as a protective factor against stress. Balancing between risk factors and protective factors is significant for teachers’ wellbeing [75].

## 5. Strengths & Limitations

The author who conducted and analysed the data worked as an early years teacher. She was also trained to work with children with behaviour problems during her master’s degree and had worked with children with ADHD and Autism. Being a mother of four boys also predisposed the author to see the other side of the coin, (i.e., allowed her to understand the child’s perspective). Being a parent and a teacher provided a broad perspective that may have enriched the data by co-constructing the reality between her knowledge and the teachers’ views.

Previous research has stressed the need for qualitative research to explore teachers’ experience of working with children with ADHD symptoms to learn about the barriers and the facilitators in providing those teachers and children with better services in the UK. The participants in this analysis were all certified teachers, teaching assistants or SENCos. Strong themes were identified and no new patterns were evident in the later interviews suggesting data saturation [46]. Furthermore, themes were compatible with quantitative and qualitative findings form other studies in the literature, supporting their validity.

This study has few potential limitations that ought to be considered when interpreting these findings. Eight of the participants worked at the same (Muslim) faith school, which was because the gatekeeper in this school was particularly supportive of the study. However, it should be noted that most of the teachers from that school were native English speakers. The lack of demographic data is considered as a limitation; this was intentionally implemented to provide teachers with a sense of privacy and enable them to express themselves freely during the interview. Another limitation was that only one of the sample was male which can potentially be explained by the fact that only 15 per cent of primary school teachers in the UK are males [76]. Repeating the research with a sample of male teachers would allow contrast of perspectives between genders.

## 6. Conclusions

In conclusion, this study provides an in-depth insight into teachers’ experiences with children with ADHD symptoms. The findings from this study suggest teachers are struggling to support children with ADHD symptoms, which can have a detrimental impact on both teachers and children due to a lack of resources and training. Teachers require more training in managing externalising behaviour in the classroom. Teachers also prefer a source of education that can be used in their spare time. Future research should focus on the lack of teacher-related interventions regarding classroom management of children with ADHD symptoms. An important focus would be the impact of the intervention on the teacher-child relationship across different settings and cultures [77,78]. This may assist in understanding cultural nuances and tailoring interventions appropriately [38]. It would be important to explore the impact of improving teachers’ coping on classroom management, teacher-child relationships, and children’s externalising behaviour.

Interventions to help teachers support children with symptoms of ADHD are likely to improve outcomes for children and teachers and improve the teacher-child relationship. Enhancing teachers’ skills in management of ADHD in the early school years could help create a protective cycle of reduction in disruptive child behaviours, lower levels of teacher stress and a more responsive teaching style.

## Data Availability

Data is available on request due to privacy and ethical restrictions. The data presented in this study are available on request from the corresponding author R.A.
